# Antinociceptive and Anti‐inflammatory Effect of Nicandrin B Isolated From *Datura ferox* in Zebrafish

**DOI:** 10.1002/cbdv.202501221

**Published:** 2025-07-05

**Authors:** Jéssica Bezerra Maciel, Hortência Ribeiro Liberato, Antônio Wlisses da Silva, Maria Auxiliadora Solange Silva Gondim Pereira, Emanuela de Lima Rebouças, Francisco das Chagas L. Pinto, Matheus Nunes da Rocha, Maria Kueirislene Amâncio Ferreira, Janaina Serra Azul Monteiro Evangelista, Izabel Florindo Guedes, Marcia Machado Marinho, Emmanuel Silva Marinho, Andreia Ferreira de Castro Gomes, Otília Loiola Pessoa, Jane Eire Silva Alencar de Menezes, Hélcio Silva dos Santos

**Affiliations:** ^1^ Graduate Program in Natural Sciences State University of Ceará Fortaleza Brazil; ^2^ Department of Organic and Inorganic Chemistry Federal University of Ceará Fortaleza Brazil; ^3^ Laboratory of Comparative Experimental Morphology – FAVET Faculty of Veterinary Medicine State University of Ceará Fortaleza Brazil; ^4^ Laboratory of Biotechnology and Molecular Biology Health Sciences Center State University of Ceará Fortaleza Brazil; ^5^ Department of Biology, Environmental Biology, School of Sciences University of Minho Braga Portugal; ^6^ Center for Exact Sciences and Technology State University of Vale do Acaraú Sobral Brazil

**Keywords:** analgesia, inflammation, receptors, TRPA1 channels, TRPV1 channel, vitanolide

## Abstract

Nicandrin B (Nic B), a withanolide isolated from *Datura ferox* leaves, was investigated for its antinociceptive and anti‐inflammatory effects in adult zebrafish (Danio rerio). Animals treated with Nic B (4, 20, and 40 mg/kg) showed no toxicity and maintained normal locomotor activity. The compound significantly reduced nociception induced by formalin and hypertonic saline; these effects were reversed by TRPA1 and transient receptor potential vanilloid 1 antagonists, indicating neuromodulation of these targets. Additionally, Nic B attenuated carrageenan‐induced abdominal edema, reduced neutrophil recruitment, and decreased hepatic reactive oxygen species levels. Docking analysis confirmed a favorable binding affinity with TRPA1, supporting its therapeutic potential. These findings suggest that Nic B combines analgesic, anti‐inflammatory activity, and toxicological safety, making it a promising drug candidate.

## Graphical abstract

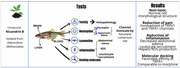

## 1 Introduction

Medications are the main form of intervention to treat health problems. However, their indiscriminate use can generate risks, especially in prolonged treatments. In the context of chronic pain, Mullachery et al. [1] report that approximately 37% of Brazilians over 50 years old use opioids for pain relief, which reinforces the need for safer and more effective therapeutic alternatives.

Pain is recognized as a relevant public health problem due to its socioeconomic impact and human suffering, affecting approximately 12%–55% of the population daily. Inflammation is present in several diseases and represents a major challenge for health systems, generating high treatment costs. Although research has advanced, there are still gaps in understanding its pathogenesis, which hinders more accurate diagnoses and treatments. Pain and inflammation are directly related, as inflammatory mediators sensitize nociceptors, making them more responsive. When stimulated, these nociceptors interact with the environment and release pro‐inflammatory substances such as prostaglandins, prostacyclins, leukotrienes, and thromboxanes. These mediators reduce the activation threshold of receptors and amplify the pain response. The contact of these substances with their specific receptors triggers biochemical cascades that transmit the painful stimulus to the central nervous system. As a result, pain becomes one of the main signs of inflammation [2, 3, 4].

In this sense, the search for new approaches in drug development has led to a growing interest in natural products, recognized for their therapeutic potential and lower incidence of adverse effects. Among these compounds, withanolides stand out due to their peculiar chemical structure, which includes a lactone in the side chain and confers diverse pharmacological properties. Studies have already demonstrated their anticancer potential [5], antinociceptive and anti‐inflammatory potential [6], in addition to their immunosuppressive action [7]. However, research related to the pharmacological and biological activities of Nicandrin B (Nic B), isolated from *Datura ferox*, is not very in‐depth, opening up space for the development of new studies [8].

However, for these natural compounds to be validated and potentially applied in the clinic in the future, a rigorous research process is necessary, including the evaluation of their efficacy and safety. At this stage, animal models play an essential role, allowing the confirmation of the biological effects of these substances. Traditionally, rodents represent about 90% of the models used in laboratories, but new alternatives have been gaining ground. Among these alternatives, zebrafish has stood out as a promising model due to its small size, low maintenance cost, rapid reproduction, and high genetic homology with humans, estimated at approximately 70%. [9]

Therefore, this study investigated the toxicity, locomotor activity, antinociceptive, and anti‐inflammatory effects of Nic B, isolated from *D. ferox* in adult zebrafish (Danio rerio). In addition, the possible mechanisms of action involved in these processes were explored, focusing on the modulation of TRPA1 and TRPV1 channels.

## 2 Results and Discussion

### 2.1 96 h Toxicity Test

Nic B (4; 20 and 40 mg/kg; 20 µL; i.p.) was not toxic to ZFa, as it did not cause death during the 96 hours of follow‐up after treatment with the sample at the three doses (LD_50_ > 40 mg/kg), as well as there was no change in the locomotor activity of the animals observed in the open field test.

### 2.2 Locomotor Activity

**FIGURE 1:**
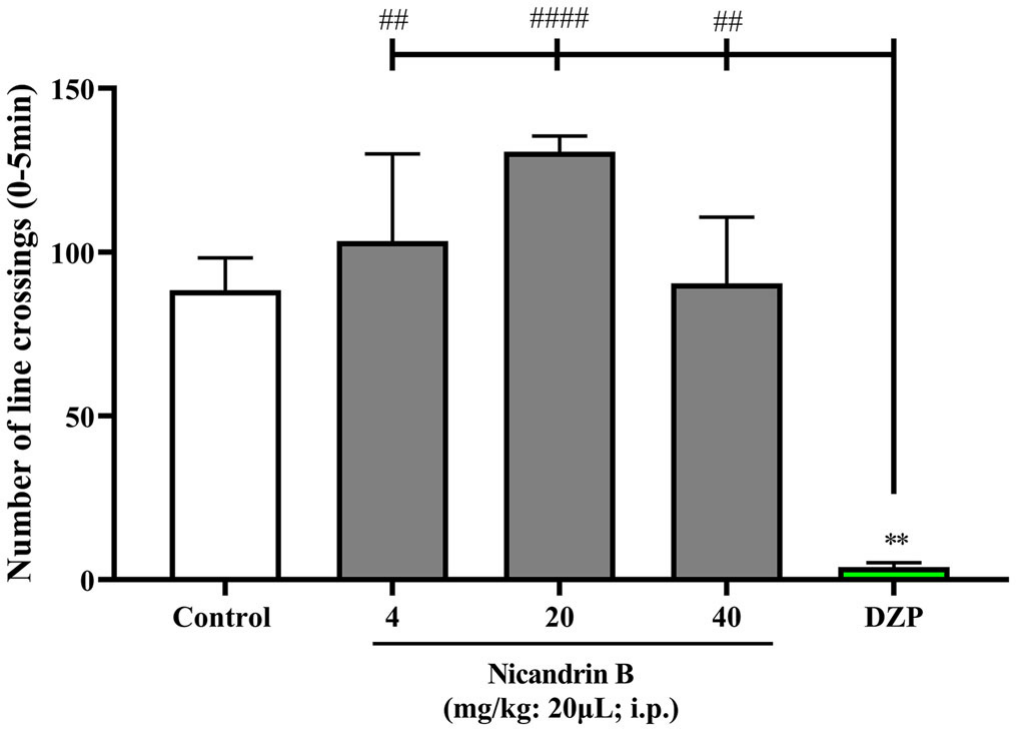
Effect of Nicandrin B on locomotor behavior of adult zebrafish in the Open Field Test (0–5 min). Values represent the mean ± standard error of the mean for six animals/group; analysis of variance (ANOVA) followed by Tukey's test. (**p < 0.01 vs. Control, ##*p <* 0.01, ####*p <* 0.0001 vs. DZP).

The open field test was performed to assess whether the sample could induce changes in the motor coordination of the animals [10], either due to an anxiolytic effect and/or muscle relaxation. The results demonstrated that the sample did not affect the locomotion of the animals (Figure 1), since there was no significant difference between all doses of Nic B and the positive control (Diazepam), which, in this case, promotes lethargy in the animals. This finding reinforces the pre‐clinical safety of the tested substance and indicates the absence of a sedative effect, which is fundamental, considering that subsequent analyses involve antinociceptive tests using the open field, in which the sample cannot interfere with this behavior.

### 2.3 Antinociceptive Activity

**FIGURE 2:**
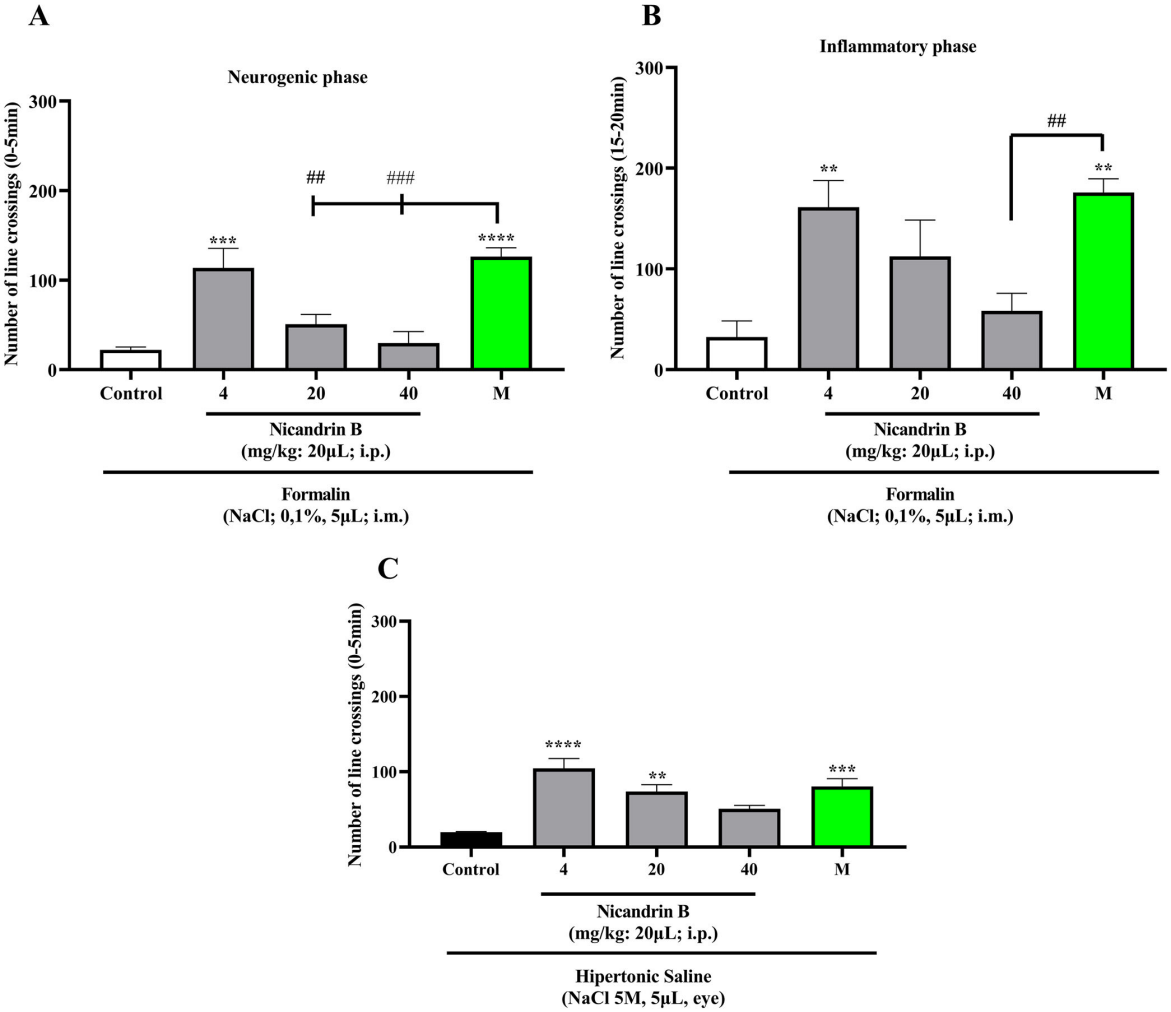
(A) Effect of nicandrin B on formalin‐induced nociception on the neurogenic phase in adult zebrafish. (B) Effect of nicandrin B on formalin‐induced nociception on the inflammatory phase in adult zebrafish.(C) Effect of nicandrin B on hypertonic saline‐induced nociception in adult zebrafish. Each column represents the mean ± standard error of the mean (*n* = 6 groups). Control: vehicle (dimethyl sulfoxide [DMSO] 3.0%; 20 µL, i.p.). Morphine (8.0 mg/kg; 20 µL; i.p.). One‐way analysis of variance (ANOVA) followed by Tukey's test: (**p < 0.01; ***p < 0.001 ****p < 0.0001 vs. Control; ##*p <* 0.01; ###p 0.001 vs. Morphine).

Statistical analysis by one‐way ANOVA, followed by Tukey's test, revealed that the lowest dose of Nic B (4 mg/kg; 20 µL; i.p.) significantly reduced formalin‐induced nociception in the neurogenic and inflammatory phases (**p < 0.01, ***p < 0.001 vs. Control; Figures 2A and 2B). These data indicate pain inhibition in both phases tested, neurogenic and inflammatory formalin, suggesting that the substance may act on both peripheral and central pain mechanisms, according to the study by [11], in which pain reversal was observed in both phases of the formalin effect in adult zebrafish.

In addition, the lower and intermediate doses (4 and 20 mg/kg; 20 µL; i.p.) significantly inhibited hypertonic saline‐induced nociception (**p < 0.01, ****p < 0.0001 vs. Control; Figure 2C). This effect was similar to that observed in the group treated with morphine, which also promoted analgesia in the animals (**p < 0.01, ***p < 0.001, ****p < 0.0001 vs. Control). The activation of TRPV1 channels present in the cornea triggers a cascade of stimuli that increases pain sensitivity, contributing to hyperalgesia. When hypertonic saline comes into contact with the cornea, it generates osmotic stress, leading to dehydration of epithelial cells, possibly due to the high concentration of sodium present in the solution. This stress activates TRPV1 channels, allowing the entry of calcium, for example, and the release of neurotransmitters that signal pain. Furthermore, this activation may be associated with inflammatory processes, further amplifying the painful sensation.

The simultaneous positive result in the formalin‐ and hypertonic saline‐induced nociception models indicates the presence of a multimodal effect of this substance. This suggests that it acts on different receptors, promoting analgesia by blocking multiple pain signaling pathways. Thus, the substance has a more comprehensive effect, which may make it a promising and effective option in therapies for pain relief [12].

**FIGURE 3:**
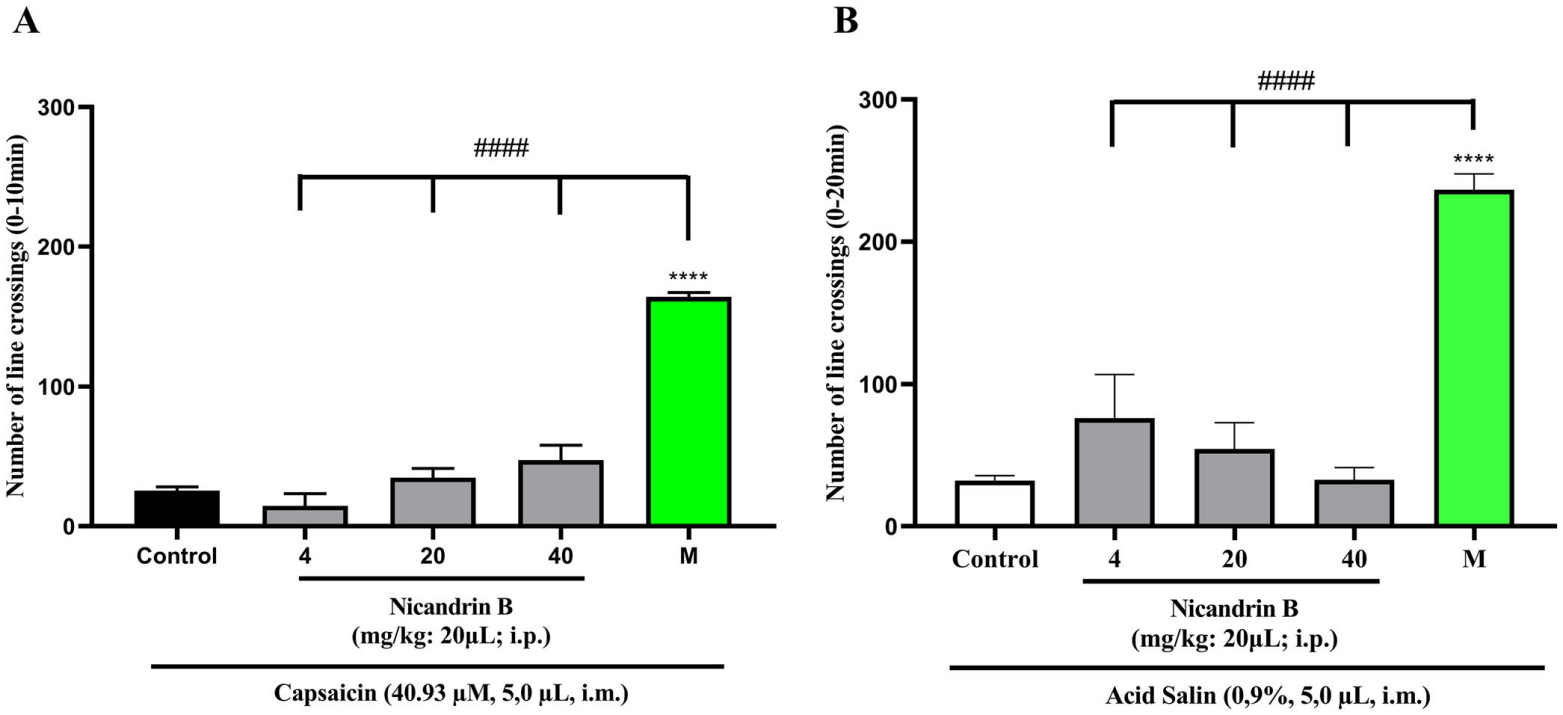
(A) Effect of nicandrin B on capsaicin‐induced nociception in adult zebrafish. (B) Effect of nicandrin B on acid saline‐induced nociception in adult zebrafish. Each column represents the mean ± standard error of the mean (*n* = 6 groups). Control (dimethyl sulfoxide [DMSO] 3.0%; 20 µL, i.p.). Morphine (8 mg/kg; 20 µL; i.p.). One‐way analysis of variance (ANOVA) with Tukey's test (****p < 0.0001 vs. Control).

On the other hand, Nic B was not able to reverse the nociceptive behavior induced by capsaicin and acid saline models (Figure 3A,B) applied to the tail. After the administration of these noxious agents, the motor impairment of the animals persisted (*p* > 0.0001 vs. Morphine), unlike what occurred in the groups treated with morphine (positive control), in which there was a significant blockade of nociception induced by capsaicin and acid saline.

### 2.4 Mechanism of Action of Nic B Induced by Formalin and Hypertonic Saline

**FIGURE 4:**
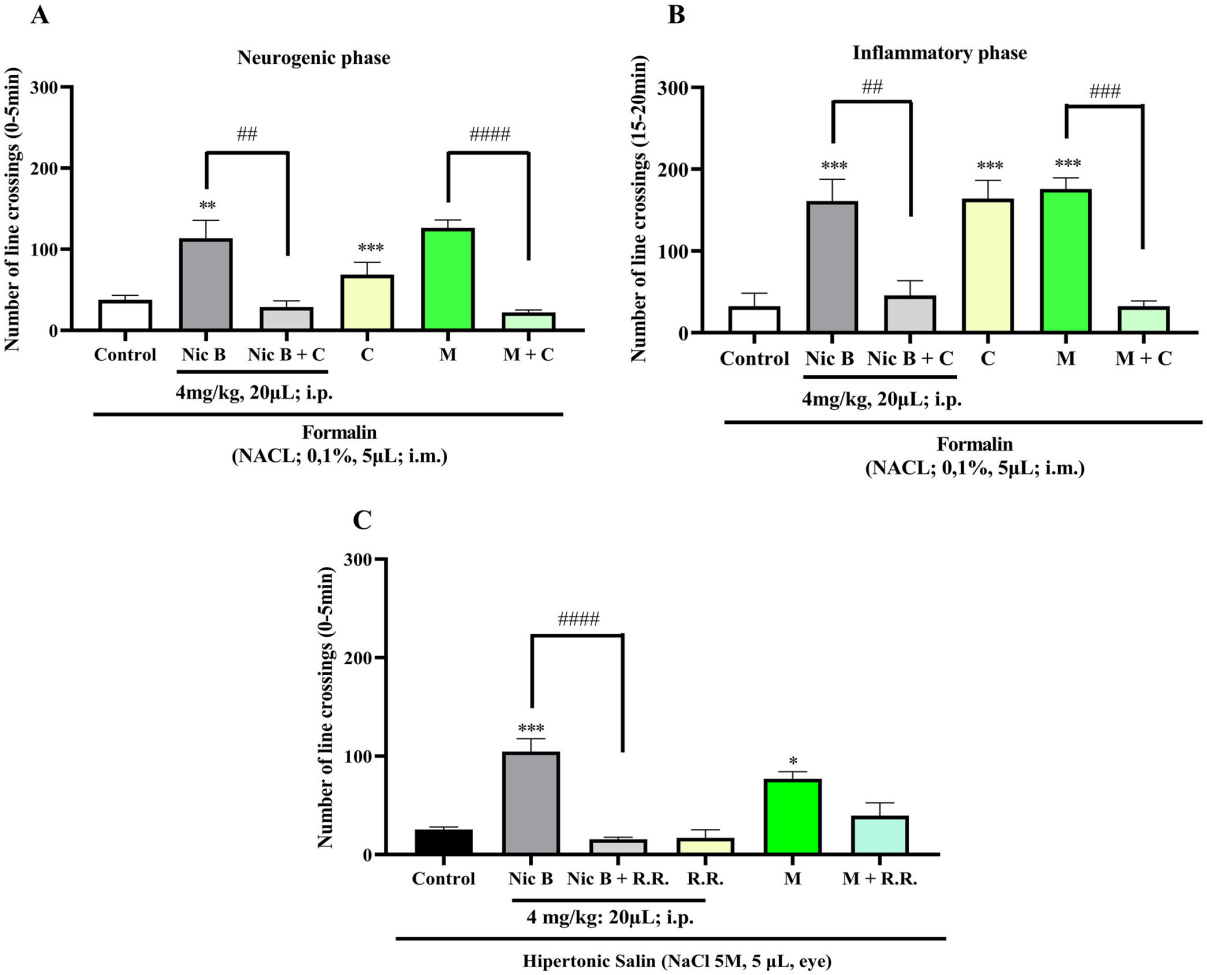
(A) Effect of nicandrin B on formalin‐induced nociception on the neurogenic phase. (B) Effect of nicandrin B on formalin‐induced nociception on the inflammatory phase. (C) Effect of nicandrin B on hypertonic saline on the antinociception of Nicandrin B (Nic B) in adult zebrafish. The column represents the mean ± standard error of the mean (*n* = 6/group). One‐way analysis of variance (ANOVA) followed by Tukey. (**p < 0.01; ***p < 0.001; ****p < 0.0001 vs. control; ##*p <* 0.01 vs. Nicandrin B vs. Nicandrin B + Camphor, Neurogenic phase; ##*p <* 0.01 vs. Nicandrin B vs. Nicandrin B + Camphor, Inflammatory phase; ###*p <* 0.001, ####*p <* 0.0001 Morphine vs. Morphine+Ruthenium red, Control (dimethyl sulfoxide [DMSO] 3%); Ruthenium red 12 mg/kg, 20 µL, i.p.; Camphor 30.4 mg/kg, 20 µL; i.p.).

The analgesic mechanism of action of Nic B was evaluated using Formalin (TRPA1 receptor activator) and Hypertonic Saline (TRPV1 channel activator) models, using specific antagonists. Camphor (TRPA1 receptor antagonist) was used in the neurogenic and inflammatory phases, while ruthenium red (TRPV1 channel antagonist) was used to investigate its action. The lowest effective dose of Nic B (4 mg/kg; 20 µL; i.p.) was selected for these studies. As a result, its antinociceptive effect was blocked by camphor in both phases (##*p <* 0.01 vs. Nic B vs. Nic B + Camphor (Formalin model), Neurogenic phase, Figure 4A; ##*p <* 0.01 vs. Nic B vs. Nic B + Camphor, Inflammatory phase, Figure 4B) and by ruthenium red (Hypertonic saline model) (###*p <* 0.001, ####*p <* 0.0001 Morphine vs. Morphine + Ruthenium red, Figure 4C). Furthermore, the antagonists did not affect locomotion or induce muscle relaxation in the animals.

Transient receptor potential (TRP) channels form a class of non‐selective cationic channels, generally located in the cell membrane. Among its various members, TRPA1 and TRPV1 (transient receptor potential vanilloid 1) channels stand out, playing fundamental roles in the transmission of sensory stimuli, especially those related to pain and inflammation [13].

TRPA1 channels play a crucial role in mediating pain, especially inflammatory pain. Garrison and Stuck [14] suggest that the antinociceptive activity of TRPA1 is directly associated with inflammatory responses, as this channel is activated by molecules involved in inflammatory signaling. This relationship was reinforced by Agostinho et al. [15], who evaluated the effects of the decoction of Plectranthus ornatus in a zebrafish model pre‐treated with formalin, a known pain inducer. The results indicated the activation of TRPA1, accompanied by a significant reduction in pain and abdominal edema induced by κ‐carrageenan, evidencing the participation of this channel in inflammatory processes.

Regarding Nic B and its interaction with TRPV1 channels through hypertonic saline, it is worth noting that TRPV1 is a pro‐nociceptive ion channel involved in the modulation of inflammatory pain. Its sensitization can intensify the release of inflammatory mediators associated with nociception [16]. A study by Magalhães et al. [17], when analyzing a corneal nociception model in zebrafish, demonstrated that this species has high representativeness in experimental tests due to its genetic homology with humans. The confirmation of this experimental applicability was evidenced by the increase in nociceptive behavior after the application of the hypertonic saline agonist, by the inhibition of this effect through the blockade of the channel with naloxone, and by the significant attenuation of nociception in response to the administration of morphine.

These findings are in agreement with the results of the present research, in which Nic B not only presented an antinociceptive effect, but also demonstrated the ability to reduce the inflammatory process, as evidenced in the subsequent results, strengthening the correlation between the activation of TRPA1 and TRPV1 channels in the regulation of the inflammatory response, highlighting their role in analgesia and inflammation control.

### 2.5 Anti‐inflammatory Activity

**FIGURE 5:**
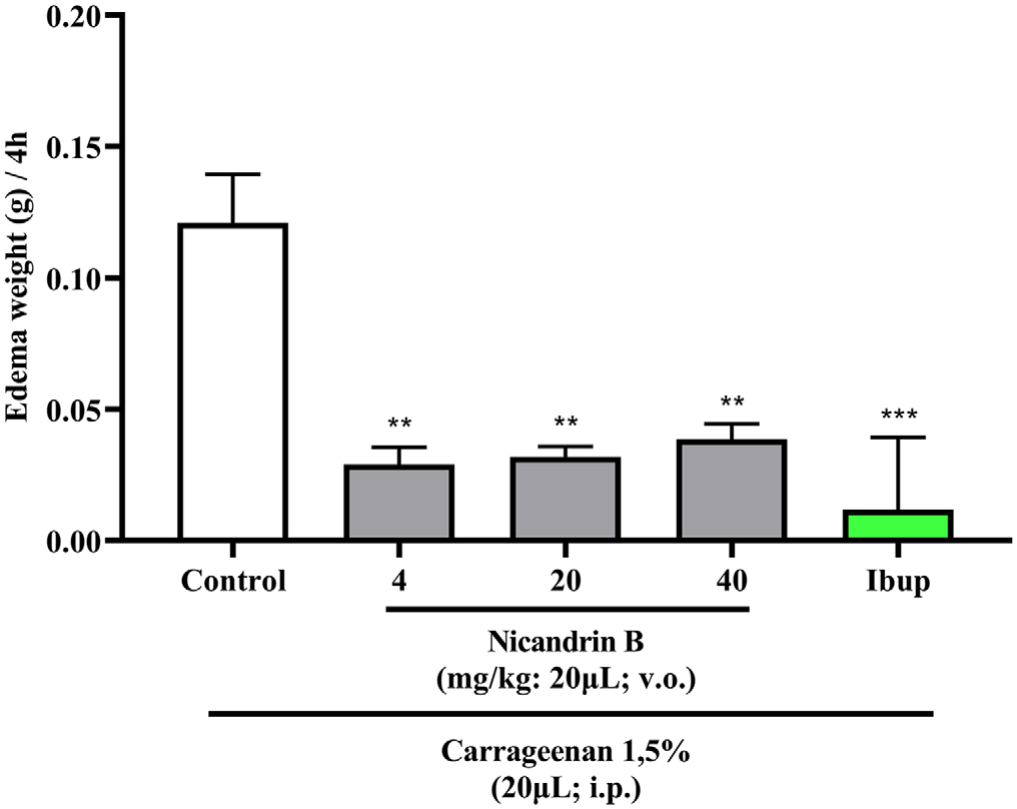
Effect of Nicandrin B on abdominal edema induced by 1.5% κ‐carrageenan in adult zebrafish, analyzed over a 4‐h period. Each column represents a mean ± standard error of the mean (*n* = 6/fish). One‐way analysis of variance (ANOVA) with Tukey's post‐hoc test (**p < 0.01 vs. control).

The one‐way Anova statistical analysis indicated that pre‐treatment with Nic B significantly reduced (**p < 0.01 vs. control) abdominal edema induced by carrageenan, similarly to what occurred with the ibuprofen positive control group (***p < 0.001 vs. control) and was significantly different from the 3% DMSO group—negative control (Figure 5), indicating the anti‐inflammatory effect of Nic B at all tested doses.

Inflammation was induced by applying 1.5% κ‐carrageenan to adult zebrafish, which allows the release of inflammatory mediators, resulting in the formation of abdominal edema. The study by Huang et al. [18] used the zebrafish model to evaluate edema induced by λ‐carrageenan, demonstrating efficient results in inducing inflammation, with methylprednisolone being able to inhibit the inflammatory response. Treatments with anti‐TNF‐α antibody and iNOS inhibitor also reduced abdominal edema, highlighting the zebrafish model as an effective tool for screening anti‐inflammatory drugs. These data corroborate the results obtained in the present research, which show that Nic B has an anti‐inflammatory effect, reducing abdominal edema induced by carrageenan.

### 2.6 Differential Leukocyte Count

Percentage distribution of leukocytes in zebrafish with abdominal edema induced by 1.5% κ‐carrageenan, treated with 3% DMSO, ibuprofen (Ibu) or Nic B (4 mg/kg) represented by Table 1, and the graph showed an increase in the number of neutrophils in the animal, which confirms the activation of the immune system against the acute inflammatory process in the negative control group (3% DMSO) induced by κ‐carrageenan, and relatively similar numbers for the positive control (ibuprofen) and test sample (Nic B).

**TABLE 1:** Differential leukocyte counts (%) in zebrafish with abdominal edema induced by κ‐carrageenan 1.5%. Values are expressed as mean ± standard error of the mean (SEM), accompanied by the range of variation for each leukocyte type (neutrophils, monocytes, lymphocytes, and eosinophils) (*n* = 6 groups).

	DMSO 3%		Nicandrin B (4 mg/kg)		Ibu	
	Mean ± SEM	Range	Mean ± SEM	Range	Mean ± SEM	Range
Neutrophilis	67 ± 2,23	60 ‐ 76	45 ± 4,02	32 ‐ 56	43 ± 3,57	32 ‐ 52
Monocytes	16 ± 6,5	6 ‐ 22	22 ± 3,2	14 ‐ 34	29 ± 12,0	20‐ 42
Lymphocytes	14 ± 5,7	8 ‐ 22	31 ± 12,5	22 ‐ 38	22 ± 9,1	14 ‐ 32
Eosinophils	2 ± 0,8	0 ‐ 2	2 ± 0,8	0 ‐ 2	2 ± 0,8	0 ‐ 2

Just as in humans, zebrafish are capable of activating immunological responses to inflammatory processes. Among the main cells involved in this mechanism, neutrophils stand out for being the most abundant in this animal model and the first to be recruited during inflammation [19].

**FIGURE 6:**
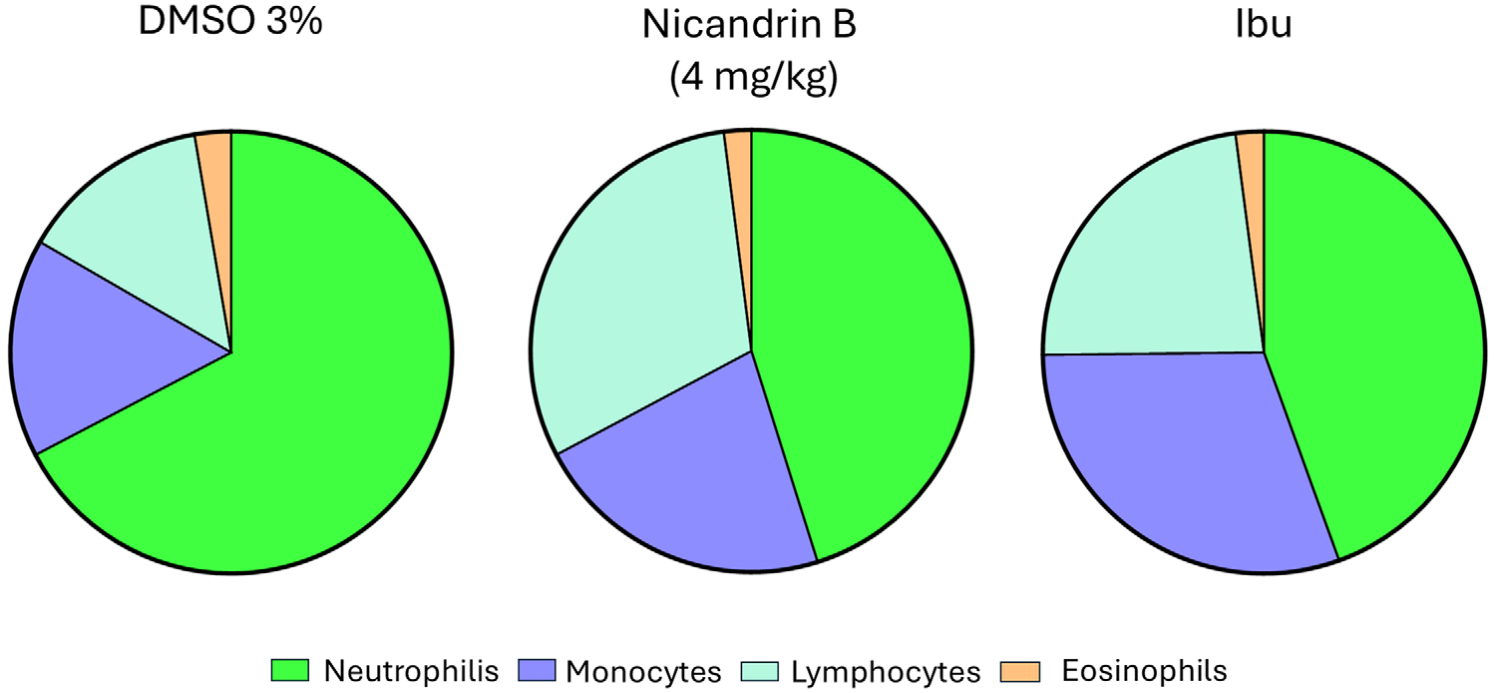
Differential leukocyte counts (%) in zebrafish with abdominal edema induced by κ‐carrageenan 1.5%. Pie charts show the relative proportion of neutrophils (green), monocytes (blue), lymphocytes (purple), and eosinophils (orange) in each experimental group (*n* = 6 groups).

In this context, the differential leukocyte count presented in Table 1 and Figure 6 reveals a significant increase in the number of circulating neutrophils in the negative control group (DMSO 3%), suggesting the migration of these cells to other tissues in response to the intraperitoneal inflammatory process caused by the edema induced by κ‐carrageenan 1.5%. On the other hand, the group treated with Nic B and the positive control (Ibuprofen) maintained reduced and similar values for neutrophils and other cells in the leukogram, suggesting a possible normalization and/or reduction of the acute inflammatory process.

The relevance of the differential leukocyte count as a tool for assessing the immunological response in zebrafish was reinforced by Grzelak et al. [19], who demonstrated its application in the investigation of acute and chronic stress in this experimental model, highlighting its potential for mapping immunological mechanisms in the species.

## 3 Histopathology in Whole Zebrafish Treated With κ‐Carrageenan 1.5%

Histopathological analysis of whole zebrafish, performed four hours after induction of abdominal edema with 1.5% κ‐carrageenan, revealed that in the negative control group (animals treated only with 1.5% κ‐carrageenan), organs such as the liver and intestine showed preserved tissue architecture. Similarly, in the positive control group (animals treated with 1.5% κ‐carrageenan and ibuprofen), the histological organization of the organs was maintained, with the presence of circular collagen fibers in specific regions of the liver and in the serosa layer of the intestine.

**FIGURE 7:**
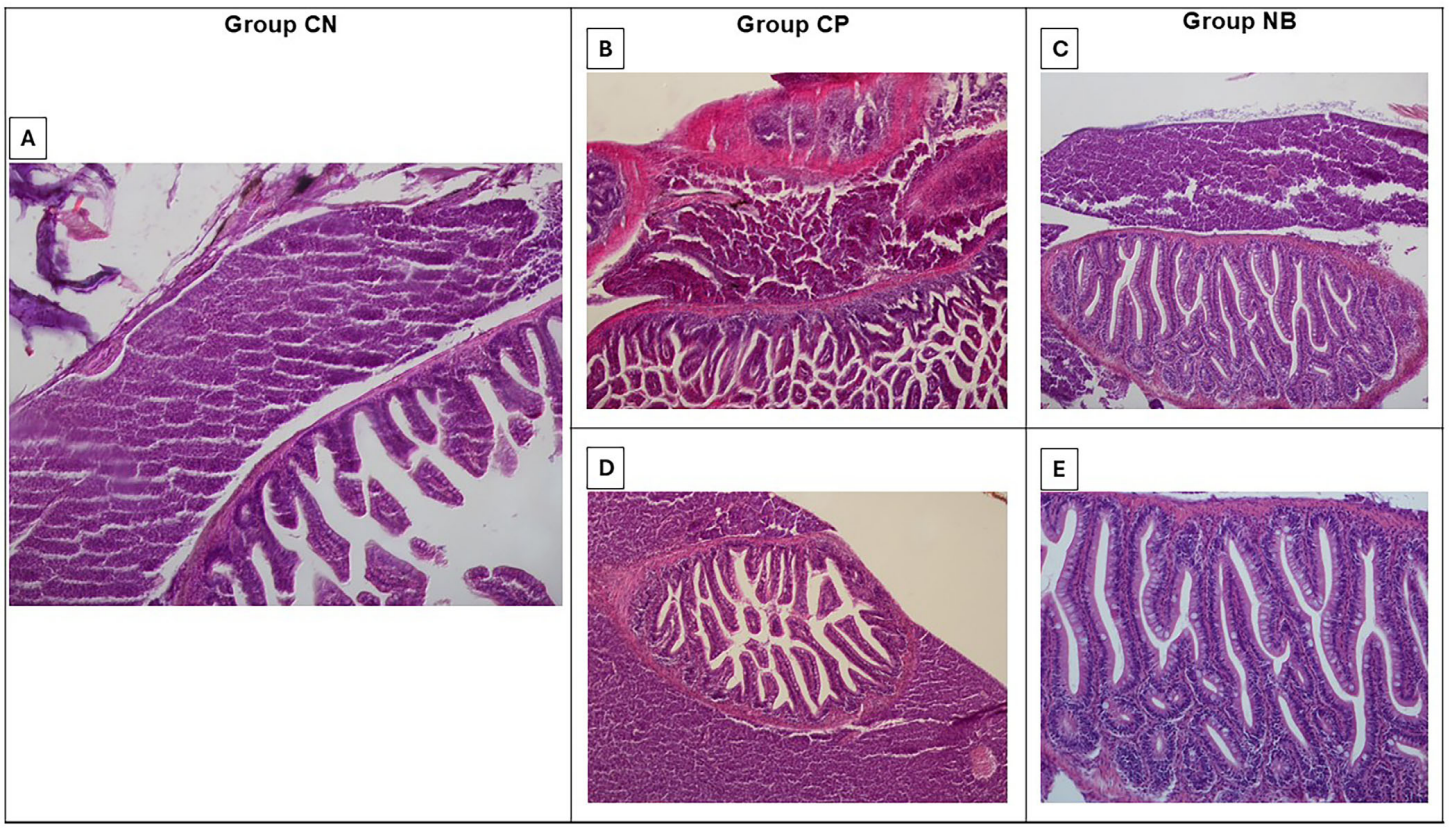
Photomicrograph of Zebrafish Liver and Intestine. (A) Negative Control Group—NC: Liver and Intestine. Organs with preserved tissue architectures in a panoramic view (H.E./10x); (B) Positive Control Group—PC: Liver and Intestine with collagen fiber deposition in the liver (*) (H.E./10x); (C) Nicandrin B Group—NB: Liver and Intestine. Discrete presence of congested vessels in the liver (**) (H.E./10x); D) Positive Control Group—PC: Liver and Intestine. Delimitation by collagen fibers in the serosa layer of the intestine (H.E./10x); (E) Nicandrin B Group—NB: Zebrafish Intestine. Highlighted collagen fibers were observed in the serosa layer of the intestine (H.E./20x). Liver (wide arrow)/Intestine (thin arrow). Trinocular Nikon Microscope/Nis 4.0 software.

In the group treated with Nic B (animals that received 1.5% κ‐carrageenan and Nic B), structural preservation of the tissues was also observed, with congested vessels in delimited areas of the liver. In addition, the presence of well‐stained collagen fibers (H&E) in the serosa layer of the intestine was evident (Figure 7). Figure 7 highlights the well‐defined histological architecture of the intestinal villi and Lieberkühn glands.

No evident signs of inflammation were identified in the microscopic analysis, nor moderate to intense vascular congestion, leukocyte infiltrate, hemorrhage, or necrosis. The absence of visible changes by optical microscopy may be related to the short analysis period (4 h). According to Richardson et al. [20], studies on cutaneous wound healing in zebrafish suggest that tissue regeneration can occur normally, even in the absence of a robust inflammatory response in the initial stages. This characteristic may be partially applicable to liver regeneration, a more complex process, in which inflammation tends to be more pronounced in advanced phases, such as tissue remodeling and cell recruitment [21].

Despite these considerations, the photomicrographs demonstrated the absence of toxicity in the tissue architecture of the tested samples, as no significant structural or morphological changes were observed in the tissues analyzed by optical microscopy (Figure 7).

**FIGURE 8:**
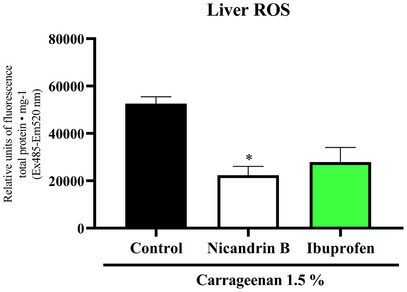
Effect of Nicandrin B on hepatic oxidative stress induced by abdominal edema in adult zebrafish. Values represent the mean ± standard error of the mean (SEM) for six fish/group (three animals in duplicate). One‐way analysis of variance (ANOVA) followed by Tukey (*p < 0.05 vs. control).

### 3.1 Oxidative Stress

Oxidative stress induced by reactive oxygen species (ROS) can trigger pathogenic effects on cellular components such as proteins, nucleic acids, carbohydrates, and lipids. Thus, its evaluation is essential to understanding abdominal edema resulting from κ‐carrageenan administration [22, 23].

One‐way ANOVA statistical analysis, followed by Tukey's test, demonstrated that the Nic B sample significantly reduced ROS levels in liver tissues compared to the control group (*p < 0.05 vs. control), an effect similar to that observed with the positive control (ibuprofen) (Figure 8). Studies such as Albarakati [24] indicate that increased ROS production is directly related to the inflammatory process induced by 1.5% κ‐carrageenan, being mediated by the activation of immune system cells and the release of inflammatory mediators.

In this context, Nic B demonstrated a protective effect against liver tissue oxidation induced by intraperitoneal administration of 1.5% carrageenan, suggesting its antioxidant and anti‐inflammatory potential.

Zebrafish have emerged as an alternative experimental model in pharmacological screening due to their high genetic homology with humans, rapid response to treatments, transparent body, and low maintenance cost. In the present study, these features enabled behavioral, inflammatory, and histological evaluation [9] of Nic B's effects. However, despite these advantages, zebrafish present important limitations, such as differences in liver metabolism, the absence of some mammalian organs, and challenges in directly extrapolating doses. Therefore, although the results indicate promising therapeutic potential for Nic B, translational application to humans requires further validation in mammalian models that offer greater physiological and metabolic similarity.

### 3.2 Molecular Docking Evaluation

**FIGURE 9:**
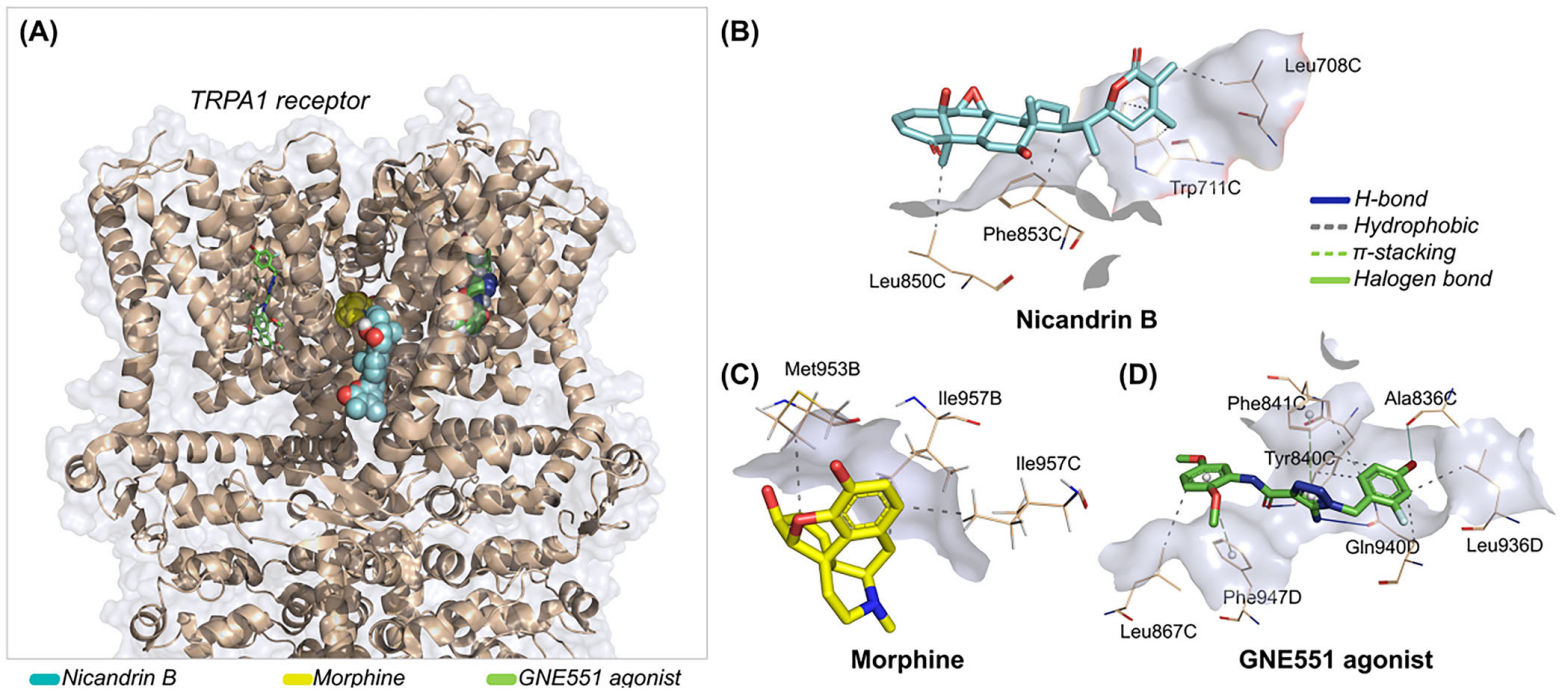
(A) three‐dimensional representation of the docking pose of the ligands nicandrin B (cyan) and morphine (yellow) in relation to the agonist GNE551 (green) on the TRPA1 receptor and three‐dimensional representation of the ligand‐receptor interactions of the ligands, (B) nicandrin B (cyan), (C) morphine (yellow), and (D) agonist GNE551 (green).

At the end of the independent molecular docking simulation cycle, it was observed that the nicandrin B ligand and the morphine control can interact with distinct protein cavities of the TRPA1 receptor, especially when compared to the GNE551 agonist, present in the membrane domain of the channel (Figure 9A). Affinity energy analysis suggests that the nicandrin B ligand has greater specificity when interacting with the TRPA1 receptor compared to the morphine control, with affinity energy values calculated at ‐7.993 and ‐7.484 kcal/mol, respectively, since both compounds are within an ideal affinity energy threshold (< ‐6.0 kcal/mol) [25]. In addition, redocking with the agonist GNE551 performed with an RMSD in the order of 1.897 Å, indicating that the reproducibility of the simulations obtained a low root mean square deviation (Table 2) [26]. The agonist showed an affinity energy calculated at ‐6.307 kcal/mol, indicating that the compounds nicandrin B and morphine have a more favorable affinity spectrum within the best‐pose selection criteria (Table 2).

**TABLE 2:** Data from molecular docking simulations expressed in root mean square deviation (RMSD) and affinity energy, and details of ligand‐receptor interactions.

Ligand	RMSD	Affinity energy	Interactions			
			Type	Residue	Chain	Distance
Nicandrin B	1.518 Å	−7.993 kcal/mol	Hydrophobic	Leu708	C	3.71
				Trp711	C	3.95
				Trp711	C	3.66
				Leu850	C	3.76
				Phe853	C	3.50
				Phe853	C	3.47
Morphine	1.501 Å	−7.484 kcal/mol	Hydrophobic	Met953	B	3.75
				Ile957	B	3.65
				Ile957	C	3.60
GNE551	1.897 Å	−6.307 kcal/mol	Hydrophobic	Tyr840	C	3.92
				Tyr840	C	3.59
				Phe841	C	3.85
				Leu867	C	3.76
				Leu936	D	3.70
				Gln940	D	3.90
			H‐bond	Tyr840	C	2.44
				Gln940	D	2.61
			π‐stacking	Phe841	C	5.06
				Phe947	D	5.27
			Halogen bond	Ala836	C	3.49

It is worth noting that the docking poses were performed at an RMSD of less than 2.0 Å. When analyzing ligand‐receptor interactions, it was observed that the nicandrin B ligand and the morphine control can interact with amino acid residues located in chain C in the transmembrane domain (Table 2). Here, it was observed that nicandrin B formed essentially hydrophobic interactions with the aromatic side chain of Trp711 and Phe853 residues and with the aliphatic side chain of Leu708 and Leu850 residues (Figure 9B), although it does not show common interactions with the GNE551 agonist, which is complexed to the TRPA1 receptor between chains C and D, by forming H‐bond interactions with the polar portion of Tyr840 and Gln940 residues (Figure 9D) [27]. On the other hand, morphine formed hydrophobic interactions with the aliphatic side chain residues Met953 and Ile957, which are located in chain B (Figure 9C). This analysis suggests that the nicandrin B ligand and morphine may act under a synergistic effect in the inhibition of TRPA1 channels [22].

#### 3.2.1 In Silico Drug‐Metabolism and Pharmacokinetics Study

##### 3.2.1.1 Multiparameter Optimization Analysis and Parallel Artificial Membrane Permeability Assay Descriptors

According to Wager et al. [28], failed bases that are poorly lipophilic (log*P* < 3) and larger and more polar than active drugs in the central nervous system (CNS) (topological polar surface area [TPSA] > 40 Å^2^) exhibit a systematic alignment between in vitro attributes of pharmacokinetics, which include high passive permeability (Papp on the order of 10⁻⁶ cm/s), low efflux by P‐gp, and low clearance (CL) in the human liver microsome (CL < 100 mL/min/kg), related to an increase in the absorption and metabolic stability of small ligands, [29] including bioactivity attributes against GPCRs and ion channels and CNS safety [30].

**FIGURE 10:**
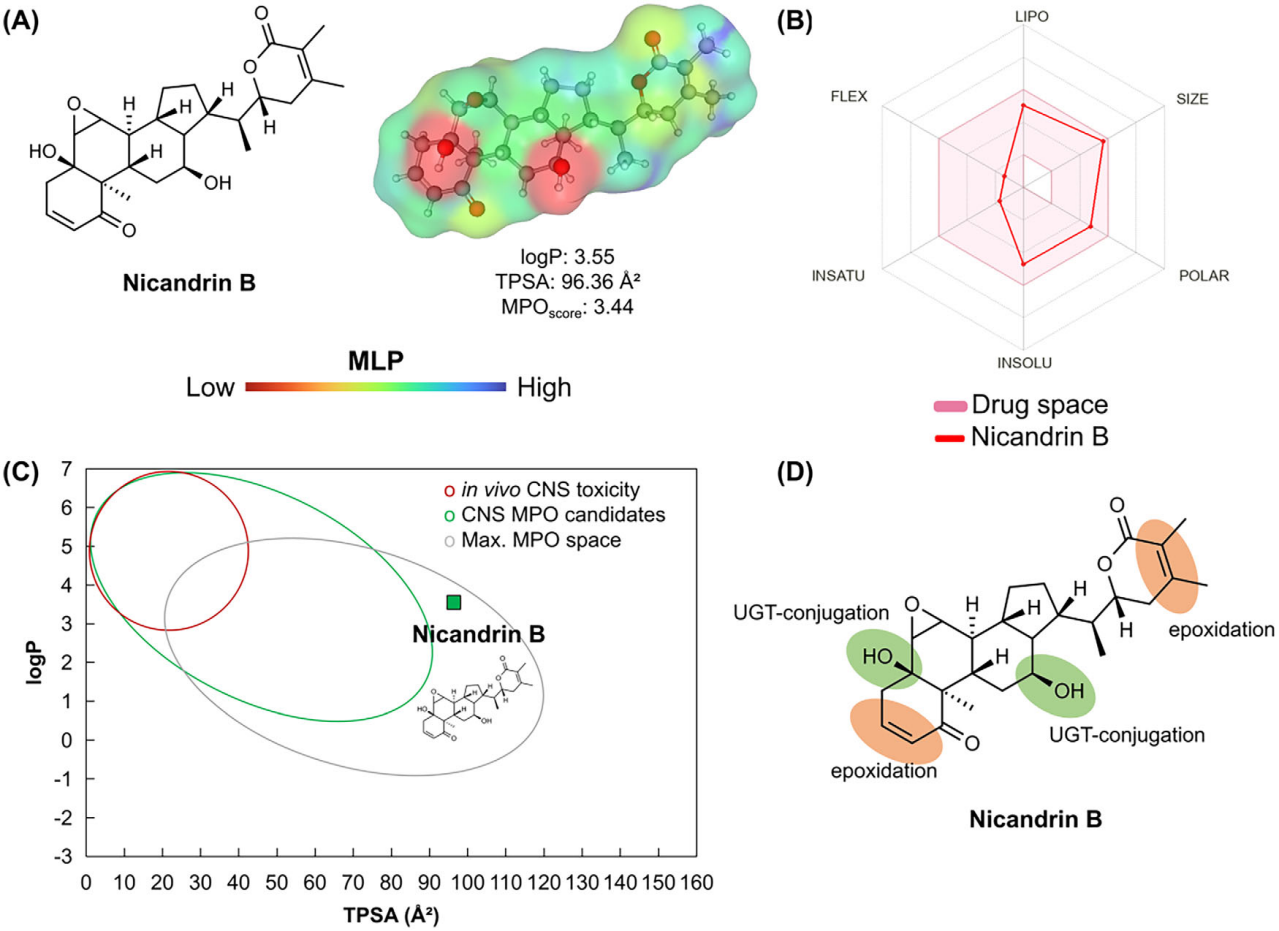
(A) surface map of molecular lipophilicity potential, (B) drug space radar that considers the ideal thresholds of lipophilicity (LIPO), size, polarity (POLAR) and solubility, (C) alignment between logP and TPSA in the prediction of the absorption spectrum and permeability in the CNS and (D) site of metabolism prediction.

A topological analysis of the molecular lipophilicity potential (MLP) can provide important information that relates lipophilicity and polarity to the permeability potential in membranes of small molecules [31, 32]. In this analysis, it was possible to observe that nicandrin B presents an essentially hydrophobic molecular surface (green to blue color spectra), with a strong contribution from the terminal methyl groups (‐CH_3_), although the hydroxyl groups (OH) exhibit high polarity (red color spectra), with a polar surface area of 40.46 Å^2^, and ester (yellow color spectra), with a polar surface area of 26.30 Å^2^ (Figure 10A) [33], resulting in a log*P* on the order of 3.55 (Table 3). The compound presented a Multiparameter Optimization (MPO) score on the order of 3.44 (on a scale of 0–6) that expresses a balance between the attributes of lipophilicity, molecular size, and polarity (Figure 10B).

**TABLE 3:** Physicochemical properties of nicandrin B calculated and applied to the Multiparameter Optimization (MPO) score system.

Property	Value	T0
logP	3.55	0.72
logD at pH 7.4	3.55	0.22
MW	470.61 g/mol	0.21
TPSA	96.36 Å^2^	0.79
HBD	2	0.50
pKa	−0.37 (basic)	1.00
MPO score	3.44	
Pfizer rule	Accepted	

When the attributes of lipophilicity and polarity (log*P* vs. TPSA) were aligned, it was possible to observe that nicandrin B resides in a physicochemical space of compounds absorbed in the human intestine and that gradually penetrate the blood‐brain barrier (BBB), especially due to TPSA > 90 Å^2^, which may reduce the cellular permeability of the compound (Figure 10C). This result agrees with the predicted descriptors of Parallel Artificial Membrane Permeability Assay (PAMPA), where the Papp on the order of 10⁻⁶ cm/s in Caco‐2 cell is an estimate of good intestinal absorption, while the Papp on the order of 10⁻⁹ cm/s in Madin‐Darby Canine Kidney (MDCK) cell expresses that BBB permeability is slow but with access to the CNS (Table 4). In agreement, a Cbrain/Cblood distribution coefficient on the order of 0.3 was predicted, indicating that the free molecular fraction in blood plasma can permeate the CNS (Table 4) [34]. However, the high probability of the compound being a substrate of P‐gp suggests passive efflux in the intestinal environment that may reduce the absorption of nicandrin B, although the predicted value of around 71% is within a threshold considered ideal [35].

**TABLE 4:** Drug‐metabolism and pharmacokinetics (DMPK) properties expressed in parallel artificial membrane permeability assay (PAMPA) and human liver microsome (HLM) stability descriptors, predicted by the PreADMET, ADMETlab, and ADMETboost tools.

Property	Value	Source
P_app_ Caco‐2	2.19 × 10⁻⁶ cm/s	PreADMET
P_app_ MDCK	5.20 × 10⁻⁹ cm/s	PreADMET
P_eff_ (P‐gp sub.)	0.948	ADMETlab
Fraction absorbed %	71.35%	PreADMET
CNS (C_brain_/C_blood_)	0.30	PreADMET
CL_int,u_	10.30 mL/min/kg	ADMETlab
CL_Hepa_	51.08 µL.min^−1^(10^6^ cells)^−1^	ADMETboost
CL_Micro_	44.57 mL.min^−1^.g^−1^	ADMETboost

##### 3.2.1.2 Human Liver Microsome Stability

Predicting the metabolic stability of a drug candidate allows us to estimate the reactivity of secondary metabolites formed in the human liver microsome (HLM) system, through phase I metabolism driven by CYP450 isoenzymes. One of these secondary metabolites is the epoxide, an intermediate in the hydroxylation of unsaturated centers, whose reactivity towards proteins and DNA can result in toxic organic response through metabolic activation [36, 37]. Prediction of the site of metabolism can also provide information on clearance in systemic circulation, which can affect oral bioavailability and half‐life of small molecules, establishing relationships between dose and toxicity [38].

With the results, it was possible to observe that nicandrin B presents cyclic unsaturated centers conjugated to carbonyl that are susceptible to hydroxylation, possibly forming unstable epoxide‐based intermediates (Figure 10D), in phase I metabolism, while the OH groups are conjugated by glucuronidation dependent on UDP‐glucuronosyltransferase (UGT), in phase II metabolism (Figure 10D). This analysis suggests that the formation of reactive metabolites is indicative of biotransformation pharmacokinetics based on the control of the administered daily oral dose [38]. In this way, a CLint,u was estimated in the order of 10.30 mL/min/kg, indicating a low clearance of the intrinsic species of nicandrin B, resulting in a viable oral bioavailability, while CLMicro = 44.57 mL min‐1 g‐1 indicates that the substance presents better stability in the microsome system (intracellular) than in the hepatic system (51.08 µL min‐1(106 cells)‐1).

## 4 Experimental

### 4.1 Characterization and Isolation of Nic B

**FIGURE 11:**
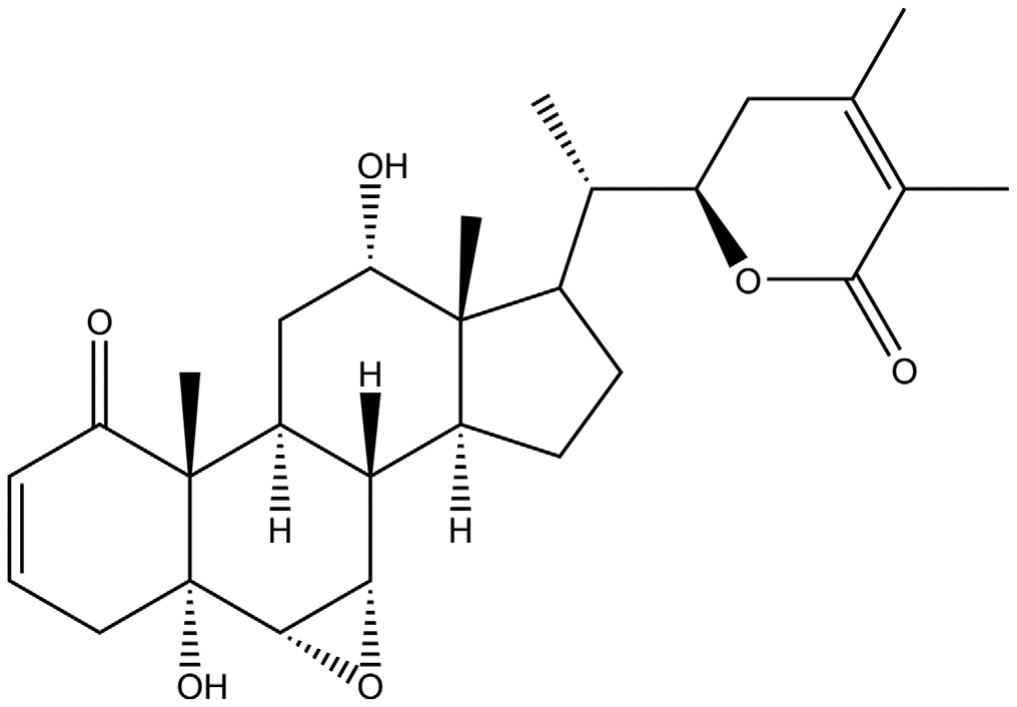
Chemical structure of vitanolide Nicandrin B.

The vitanolide Nic B (Figure 11) was extracted and isolated from dried and crushed leaves of *D. ferox*. The plant material was collected in May 2008 in the municipality of Apuiarés‐CE and cataloged under no. 42384 in the Prisco Bezerra Herbarium (EAC) of the Federal University of Ceará—UFC and registered in Sigen under number A86B918. The first characterizations were performed by Bagchi et al. [39].

RMN 1H (300 MHz em CDCl_3_); *δ* 6.60 (ddd, J = 9.9, 4.8, 1.7 Hz, H‐3), 5.82 (dd, J = 9.9, 1.7 Hz, H‐2), 4.36 (dt, 13.2, 2.8 Hz, H‐22), 4.02 (t, J = 6.0 Hz, H‐12), 3.30 (dd, J = 3.6, 1.7 Hz, H‐7), 3.03 (d, J = 3.6 Hz, H‐6), 2.84 (dt, J = 13.5, 3.0 Hz, H‐11α), 2.70 (dt, J = 18.8, 2.8 Hz, H‐4β), 2.52 (dd, J = 18.8, 5.0 Hz, H‐4α), 2.48 (dd, J = 18.4, 12.5 Hz, H‐23β), 2.26 (m, H‐9), 2.06 (m, H‐8), 2.00 (m, H‐23α), 1.95 (m, H‐20 e H‐15), 1.93 (s, H‐27), 1.87 (s, H‐28), 1.85 (m, H‐14), 1.76 (m, H‐17), 1.74 (m, H‐16β), 1.60 (m, H‐11β), 1.33 (m, H‐16α), 1.16 (s, H‐19), 1.08 (d, J = 6.0 Hz, H‐21), 0.76 (s, H‐18). Dados de RMN 13C (125 MHz em CDCl3); δ 203.7 (C‐1), 167.3 (C‐26), 149.7 (C‐24), 140.2 (C‐3), 129.0 (C‐2), 121.9 (C‐25), 78.7 (C‐22), 73.5 (C‐5), 72.5 (C‐12), 57.1 (C‐7), 56.7 (C‐6), 50.7 (C‐10), 47.1 (C‐13), 43.7 (C‐14), 42.9 (C‐17), 39.1 (C‐20), 36.9 (C‐4), 36.1 (C‐8), 29.9 (C‐23), 29.8 (C‐11), 28.9 (C‐9), 26.7 (C‐16), 23.2 (C‐15), 20.6 (C‐28), 14.8 (C‐19), 12.6 (C‐21), 12.5 (C‐27), 12.1 (C‐18).

The data of ^1^H and ^13^C nuclear magnetic resonance (NMR) are in accordance with a study by Evans et al. [39].

### 4.2 Drugs and Reagents

The drugs and reagents used in the nociception experiments were: formalin (Formaldehyde; 0.1%), acidic saline solution, hypertonic saline solution, naloxone (Tocris Bioscience), capsaicin, camphor, capsazepine, ruthenium red, and morphine obtained from Sigma‐Aldrich (Brazil), and dimethyl sulfoxide (DMSO) obtained from Cequímca. The drugs and reagents used in the inflammation experiments were: κ‐Carrageenan (Palazzo do Diet Light), ibuprofen (Advil).

### 4.3 Zebrafish

The study used adult zebrafish (*D. rerio*, ZFa) of wild strain, aged between 60 and 90 days, average weight of 4.0 ± 0.1 g and a length of approximately 3.5 ± 0.5 cm. The animals, of both sexes (*n* = 6/group), were acquired from the supplier Agroquímica: Comércio de Produtos Veterinários LTDA, located in Fortaleza, Ceará. The manipulation and acclimatization took place in the Laboratório de Bioensaios Químico‐Farmacológico e Ambiental da Universidade Estadual do Ceará (LABQFAM/UECE). The fish were kept in glass aquariums under a controlled temperature of 25°C and pH 7.0, with a 12:12 h light/dark cycle, using tap water previously treated with anti‐chlorine from the ProtecPlus brand. The environment had air pumps and submerged filters to guarantee the water quality. At the end of the experiments, the animals were sacrificed by immersion in cold water (2–4°C) until the interruption of opercular movements, according to CONCEA guidelines. All experimental procedures were approved by the Ethics Committee for the Use of Animals (CEUA) of the State University of Ceará (UECE), under protocol no. 04983945/2021.

### 4.4 General Protocol

In the experiments, zebrafish of both sexes were randomly selected and transferred to a damp sponge. Then, they received the test sample or controls intraperitoneally (i.p.) and, subsequently, were exposed to noxious agents intramuscularly (i.m.) in the tail or córnea [17, 40]. After the application of the treatments, the animals were individually allocated to glass beakers (250 mL) containing 150 mL of aquarium water and kept at rest. The intraperitoneal (i.p.), intramuscular (i.m.), and topical administrations in the cornea were performed using insulin syringes (0.5 mL; UltraFine BD) equipped with 30G needles.

The evaluations were performed by researchers blinded to the experimental conditions.

### 4.5 Evaluation of Locomotor Activity (Open Field Test)

The animals (*n* = 6/group) received intraperitoneal treatment (i.p.) with Nic B at doses of 4, 20, and 40 mg/kg (20 µL) or with the control vehicle (DMSO 3%; 20 µL; i.p.). Thirty minutes after administration, they were submitted to the open field test, being individually placed in Petri dishes (100 × 15 mm). This test aimed to verify if Nic B interferes with the motor coordination of the animals, causing sedation and/or muscular relaxation [41].

### 4.6 Acute Toxicity 96 h

After the locomotor evaluation, the fish were kept at rest and monitored for a period of 96 hours for analysis of the mortality rate. The number of deaths in each group was registered every 24 hours [42]. The lethal concentration needed to cause the death of 50% of the animals (LD_50_) was determined using the mathematical method Trimmed Spearman‐Karber, with a confidence interval of 95%.

### 4.7 Treatments

In all nociceptive tests, the animals (*n* = 6/group) were treated intraperitoneally (20 µL) with Nic B (4; 20 and 40 mg/kg), morphine (8 mg/kg—positive control), or vehicle (DMSO 3%).

In the inflammation tests, the animals (*n* = 6/group) were treated orally (20 µL) with Nic B (4; 20 and 40 mg/kg), ibuprofen (100 mg/kg—positive control), κ‐carrageenan (1.5%; 20.0 µL, i.p.—negative control) or vehicle (Control, DMSO 3%, 20 µL).

### 4.8 Nociception Behavior Induced by Noxious Agents

The animals were pre‐treated with Nic B (i.p.) 30 min before receiving the treatments with noxious stimulus (i.m.): (1) Formalin (cation channel agonist, subfamily A, member 1 [TRPA1] in neuropathic and neurogenic phases; 0.1%; 5.0 µL); (2) Capsaicin (cation channel agonist with transient receptor potential 1 of subfamily V [TRPV1]; 40.93 µM/5.0 µL); (3) Acid Saline (Acid Sensing Ion Channels [ASIC] Agonist; 0.1% acetic acid dissolved in saline solution, pH 3.28/5.0 µL). Antinociceptive activity was evaluated individually for each type of treatment. The animals were individually placed in Petri dishes (100 × 15 mm), divided into quadrants, and the nociceptive response was quantified in terms of locomotor activity, i.e., according to the number of line crossings performed during a period [41, 43, 44]. To verify the possible involvement of Nic B in the systems: TRPA1, TRPV1, and ASICs, tests were subsequently performed with antagonists of these channels [42]. The animals (*n* = 6/group) were pre‐treated intraperitoneally (5.0 µL) with naloxone (8 mg/kg, ASICs channel antagonist), camphor (30.4 mg/kg, TRPA1 channel antagonist) capsazepine (20 mg/kg, TRPV1 channel antagonist) 15 min before pre‐treatment with the lowest effective dose of Nic B (4 mg/kg i.p.). Antinociceptive activity was analyzed for each specific treatment. The animals were placed individually in a Petri dish (100 × 15 mm), divided into quadrants, and the nociceptive response was quantified in terms of locomotor activity (number of line crossings) performed during a specific period for each model described below in the results section.

### 4.9 Corneal Nociception Induced by Hypertonic Saline

The induction of nociception in the ZFa cornea was performed with hypertonic saline solution (TRPV1 agonist; NaCl 5 M; 5.0 µL) [17], applied to the right eye of the animals (*n* = 6/group) 1 h after pre‐treatment with Nic B (4, 20 or 40 mg/kg; 20 µL; i.p.) or morphine (8 mg/kg; 20 µL; i.p.; positive control) or vehicle (DMSO 3%, 20 µL; i.p.). Antinociceptive activity was analyzed in the open field test in the Petri dish as described above.

### 4.10 Edema Induction by κ‐carrageenan 1.5%

Anti‐inflammatory activity was investigated by inducing abdominal edema induced by κ‐carrageenan [23]. Animals (*n* = 6/group) received Nic B (4; 20 and 40 mg/kg; 20 µL; v.o.) or negative control (DMSO 3%; 20 µL; v.o.). A group of animals was treated with the positive control—ibuprofen (100 mg/kg; 5.0 µL; v.o.). After 1 h of treatments, the fish individually received the i.p. injection of κ‐carrageenan (1.5%; 20.0 µL). The body weight of the animals was measured before treatment and at 1 h intervals after induction of peritoneal edema over a period of 4 h. The animals were immediately sacrificed to stop the biological reactions at the end of the experiment.

### 4.11 Histopathology in Whole Zebrafish Treated With κ‐carrageenan at 1.5%

Following the acute inflammation test by edema induction by κ‐carrageenan 1.5%, the animals treated with κ‐CGN (positive control, negative control, and sample; *n* = 6/group) were euthanized by immersion in an ice bath for 10 min. Liver samples used for histological analysis were fixed in 10% formaldehyde and/or paraformaldehyde solution. After fixation, they were washed in running water, dehydrated in alcohol solutions of different concentrations, cleared in xylene, and embedded in Paraplast. Sections were performed with a thickness of 3‐5 µm, and followed by Hematoxylin‐Eosin (H.E.) staining techniques [45]. After staining the samples, the slides were analyzed and photo‐documented under a Trinocular Nikon Eclipse Ni light microscope (NIS Elements BR Software).

### 4.12 ROS Levels in Liver Tissue

To assess the levels of ROS in liver tissues of fish with edema induced by 1.5% κ‐carrageenan and treated with the negative control (3% DMSO), positive control (Ibuprofen), and sample (Nic B) groups, 2',7'‐dichlorodihydrofluorescein diacetate (DCHF‐DA) was used [46], employing the lowest effective dose in the acute inflammation test (4 mg/kg). After induction of abdominal edema with 1.5% κ‐carrageenan and administration of treatments (3% DMSO, ibuprofen, and Nic B sample), the animals (*n* = 6/group) were euthanized under ice cooling for liver extraction. Liver tissues from three animals (in duplicate) were macerated in Tris‐HCl‐EDTA buffer and then centrifuged at 10 000 × *g* for 10 min. Subsequently, 200 µL of the supernatant was collected and mixed with 5 µL of DCHF‐DA. The oxidation of DCHF‐DA into fluorescent dichlorofluorescein was used to detect the presence of ROS. The fluorescence intensity emitted by dichlorofluorescein was recorded at 520 nm (excitation at 480 nm) 2 hours after the addition of DCHF‐DA to the sample. To correlate the ROS results with the protein content of the liver tissues, protein quantification was performed using the Bradford method. Protein concentration was determined by UV‐VIS spectrophotometry at 280 nm, using a BSA standard curve [47].

### 4.13 Blood Collection and Hematological Analysis

**FIGURE 12:**
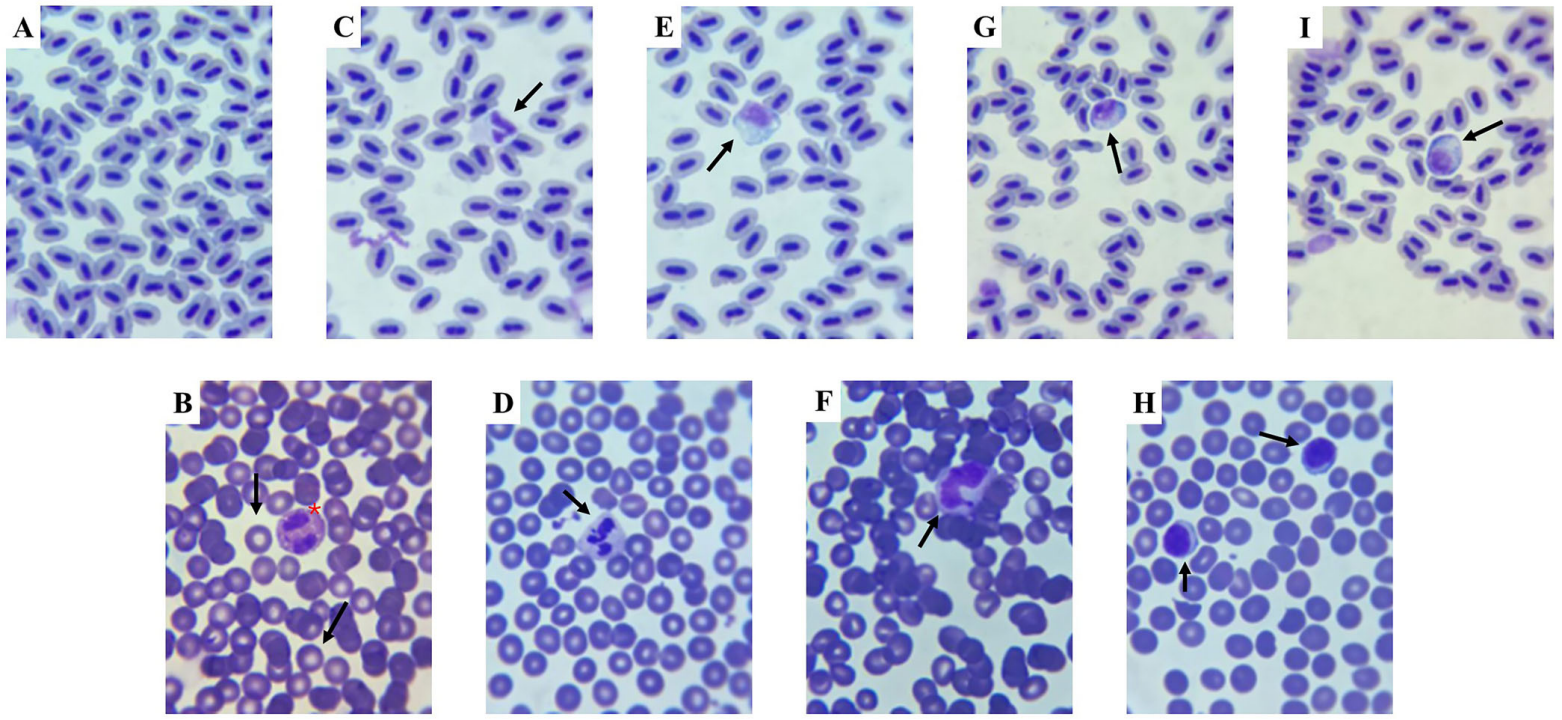
Microstructure of peripheral blood cells in adult zebrafish and humans. (A) Field with red blood cells from adult zebrafish. (B) Human red blood cells (arrows) and a human eosinophil (asterisk). (C, D) Neutrophils from adult zebrafish and humans, respectively (arrows). (E, F) Monocytes from adult zebrafish and humans, respectively (arrows). (G, H) Lymphocytes from adult zebrafish and humans, respectively (arrows). (I) Eosinophils from adult zebrafish (arrow). Staining: Fast panoptic stain.

After euthanasia of the animals, the caudal region was sectioned for immediate collection of peripheral blood (Figure 12). The blood samples were used for the preparation of blood smears, which were subsequently fixed and stained for differential leukocyte analysis. Blood smears were prepared from whole blood and stained with Quick Panoptic (Renylab, Campinas, SP, Brazil). The slides were examined by differential counting (50 cells) of leukocytes (neutrophils, monocytes, lymphocytes, and eosinophils) under oil immersion at 100 × magnification.

### 4.14 Docking Molecular

#### 4.14.1 Molecular Docking Evaluation

To evaluate the antinociceptive effect of Nic B, a series of independent molecular docking simulations were performed using the TRPA1 pathway. The ‘Structure of human TRPA1 in complex with agonist GNE551’ was retrieved from the RCSB Protein Data Bank (https://www.rcsb.org/), deposited under PDB code 6 × 2J as a membrane receptor in the organism Homo sapiens and expression system in Spodoptera frugiperda, whose three‐dimensional structure was resolved by electron microscopy at a resolution of 3.00 Å. For protein preparation, non‐polar hydrogens were removed and Gasteiger charges were computed, with a grid‐box adjusted to encompass the entire conformational space of the protein, configured with dimensions of 104 Å x 110 Å x 54 Å in the x = 164.032, y = 161.261, and z = 195.962 axes, with grid spacing in the order of 1.000 Å, using the AutoDockTools program (https://autodocksuite.scripps.edu/adt/). The AutoDockVina code (https://vina.scripps.edu/) was configured to perform a cycle of 50 independent simulations of 30 poses each from the Lamarckian Genetic Algorithm (LGA), using morphine and the agonist GNE551 as comparatives, with a degree of exhaustiveness = 64. The selection criterion for the best pose included the alignment between affinity energy (< ‐6.0 kcal/mol) and root mean square deviation (RMSD) less than 2.0 Å [48, 49]. To validate the molecular docking simulation protocol, the co‐crystallized agonist GNE551 was re‐docked and subjected to the best‐pose selection criteria.

#### 4.14.2 In Silico DMPK Study

The chemical structure of Nic B was optimized from the formalism of classical mechanics of the Merk Molecular Force Field 94 (MMFF94) method in the Avogadro2 program (https://two.avogadro.cc/) to generate the MLP map in the academic license software MarvinSketch LTS version Neon.3, Chemaxon (https://chemaxon.com/marvin). The results were related to the calculated properties of intrinsic lipophilicity (log*P*), molecular weight (MW), and TPSA. The DMPK profile was predicted by the MPO system—Pfizer, Inc.—as shown in Equation (1):

(1) $$d=\sum_{i=1}^{N}w_{k}{\left(T\left(x^{0}\right)\right)}_{k}$$

where the desirability score (d) is given by the weighting factor (w) assigned to each attribute k in its ideality thresholds (T(x)), which include the limits: logP ≤ 3, lipophilicity at physiological pH (logD at pH 7.4) ≤ 2, MW ≤ 360 g/mol, TPSA 40‐90 Å^2^, H‐bond donors (HBD) ≤ 1, and basic pKa ≤ 8 (*N* = 6 properties) [48]. The sum results in an MPO score that varies from 0 (poor DMPK) to 6 (optimal DMPK), where the results were related to the predicted descriptors of PAMPA using the PreADMET services (https://preadmet.qsarhub.com/adme/), ADMETlab (https://admetmesh.scbdd.com/) and ADMETboost (https://ai‐druglab.smu.edu/admet), which include passive cell permeability (Papp) through the cell models of Colorectal Adenocarcinoma (Caco‐2) and MDCK for estimation of intestinal permeability and the BBB, respectively, prediction of passive efflux (Peff) by P‐glycoprotein (P‐gp), fraction absorbed, and distribution in the central nervous system (CNS) [50, 51]. To estimate stability in the HLM system, a prediction of the site of metabolism was made using the XenoSite server (https://xenosite.org/), where the specificity of structural fragments is related to known substrates of cytochrome P450 (CYP450) isoforms, deposited in the server's database [36]. The results were related to the descriptors of intrinsic clearance (CLint,u) in the HLM, hepatic clearance (CLHepa), and clearance in liver microsomes (CLMicro), estimated by predicting DMPK properties.

#### 4.14.3 Statistical Analysis

The results were expressed as mean ± standard error of the mean for each group with 6 animals. After confirming the normal distribution and homogeneity of the data, the differences between the groups were submitted to one‐way ANOVA, followed by Tukey's test. All analyses were performed using GraphPad Prism v. 6.01 software. The level of statistical significance adopted was 5% (*p <* 0.05). For the leukogram, the average percentages of each type of leukocyte were entered into the GraphPad Prism graphic analysis software and Excel for the construction of the table and pie charts, using distinct colors for each cell population. The values are expressed as mean ± standard error of the mean (SEM), accompanied by the range of variation for each type of leukocyte (neutrophils, monocytes, lymphocytes, and eosinophils).

## 5 Conclusion

The results of the present study demonstrate that Nic B is a safe compound, showing no signs of toxicity in the 96‐h acute exposure tests and not inducing alterations in the locomotor activity of adult zebrafish across the behavioral models used. This safety profile was further corroborated by histological analyses, which revealed preserved tissue architecture in treated animals. Confirming the absence of toxicity allowed the progression to pharmacological evaluations of the compound. Regarding its biological activity, Nic B exhibited a promising profile as a bioactive agent, combining antinociceptive and anti‐inflammatory effects. Behavioral assessments indicated that the compound modulated TRPA1 receptor activity in the formalin‐induced nociception model and TRPV1 channels in the hypertonic saline model, suggesting a neuromodulatory mechanism of action in pain perception. These findings were reinforced by molecular docking analyses, which showed that Nic B binds to the transmembrane domain of the TRPA1 receptor at a site distinct from those occupied by the agonist GNE551 and the morphine control, suggesting potential synergistic action with other TRPA1 inhibitors and expanding its therapeutic applications. In addition to its antinociceptive activity, the compound demonstrated an anti‐inflammatory effect, evidenced by the reduction of abdominal edema induced by 1.5% κ‐carrageenan. Leukocyte count analysis revealed an increased percentage of neutrophils compared to the negative control, indicating leukocyte recruitment and activation of an acute inflammatory response. Simultaneously, Nic B also showed a hepatoprotective effect by reducing hepatic ROS levels. Furthermore, PAMPA indicated that Nic B exhibits high cellular permeability and potential access to the CNS, which may enhance its pharmacological efficacy. In conclusion, this study expands the current knowledge on natural compounds with therapeutic properties, suggesting that Nic B represents an innovative alternative for the modulation of pain and inflammation. As future directions, it is essential to validate the antinociceptive and anti‐inflammatory effects of Nic B in mammalian models, such as mice, which offer greater physiological similarity to humans. Additionally, in vivo pharmacokinetic studies are necessary to characterize key parameters such as bioavailability, half‐life, hepatic metabolism, and compound excretion, supporting the clinical development of therapeutic formulations based on this compound.

## Author Contributions

**Jéssica Bezerra Maciel**: investigation, writing, review, and editing; **Hortência Ribeiro Liberato**:supervision, formal analysis, and softwares; **Andreia Ferreira de Castro Gomes**: supervision, formal analysis, and softwares; **Antônio Wlisses da Silva**: writing the original draft and reviewing the manuscript; **Maria Kueirislene Amâncio Ferreira**: writing the original draft and reviewing the manuscript; **Emmanuel Silva Marinho**: softwares and validation; **Matheus Nunes da Rocha**: softwares and validation; **Márcia Machado Marinho**: softwares and validation; **Jane Eire Silva Alencar de Menezes**: administration and project writing; **Janaina Serra Azul Monteiro Evangelista**: administration and project writing; **Hélcio Silva dos Santos**: administration and project writing; **Maria Auxiliadora Solange Silva Gondim Pereira**: extraction, isolation, and characterization of Nicandrin B; **Francisco das Chagas L. Pinto**: extraction, isolation, and characterization of Nicandrin B; **Otília Deusdênia Loiola Pessoa**: extraction, isolation, and characterization of Nicandrin B.

## Conflicts of Interest

The authors declare no conflicts of interest.

## Data Availability

The authors have nothing to report.
